# Practical Considerations for Developing Clinical Natural Language Processing Systems for Population Health Management and Measurement

**DOI:** 10.2196/37805

**Published:** 2023-01-03

**Authors:** Suzanne Tamang, Marie Humbert-Droz, Milena Gianfrancesco, Zara Izadi, Gabriela Schmajuk, Jinoos Yazdany

**Affiliations:** 1 Division of Immunology and Rheumatology Stanford University School of Medicine Stanford, CA United States; 2 Department of Veterans Affairs Office of Mental Health and Suicide Prevention Program Evaluation Resource Center Palo Alto, CA United States; 3 Division of Rheumatology University of California, San Francisco San Francisco, CA United States

**Keywords:** clinical natural language processing, electronic health records, population health science, clinical decision support, information extraction

## Abstract

Experts have noted a concerning gap between clinical natural language processing (NLP) research and real-world applications, such as clinical decision support. To help address this gap, in this viewpoint, we enumerate a set of practical considerations for developing an NLP system to support real-world clinical needs and improve health outcomes. They include determining (1) the readiness of the data and compute resources for NLP, (2) the organizational incentives to use and maintain the NLP systems, and (3) the feasibility of implementation and continued monitoring. These considerations are intended to benefit the design of future clinical NLP projects and can be applied across a variety of settings, including large health systems or smaller clinical practices that have adopted electronic medical records in the United States and globally.

## Introduction

Natural Language Processing (NLP) has the potential to improve the delivery, quality, and safety of health care [1-7]. There have been numerous research applications, including the extraction of disorders, drugs, and procedures. Moreover, NLP methods have automated the extraction of information that is likely to be undercoded or not coded in a patient’s record, such as the severity of a disorder, their functional status, or social determinants of health [4,6-8]. However, examples of health systems operationalizing clinical NLP tools for real-world clinical decision-making, as well as population health management and quality measurement, are limited. This is a missed opportunity to turn rich, unstructured data into structured information that can be used for quality and performance initiatives within a health system or a professional field, or to make national-level comparisons [2,9-12].

To address the challenges translating research tools to clinical practice, we present practical considerations for NLP system stakeholders that can be used to position an early-stage research project for use in real-world decision-making and to eventually demonstrate institutional value. Our practical considerations are informed by prior literature and reports that describe a chiasm rather than a synergy between clinical NLP research and clinical practice. For example, Wen et al [13] share the Mayo Clinic’s Desiderata for the implementation of an NLP development delivery platform derived from 2 decades of implementing clinical NLP in their health system. Lederman et al [14] describe how existing clinical NLP systems “have delivered marginal practical utility and are rarely deployed into health care settings” and call for a new paradigm of clinical NLP research for real-world decision support. Similarly, Newman-Griffis et al [15] call for a new paradigm and general principles for clinical NLP research that are focused on challenges posed by application needs and describe how these challenges can drive innovation in basic science and technology design. Referring to artificial intelligence systems in medicine more broadly, Topol et al [16] have also observed that “deployment of medical AI systems in routine clinical care provides an important yet largely unfulfilled opportunity”. We also draw from our own collective experience developing clinical NLP systems for research studies and in an operational capacity.

Our practical considerations can be used to support the development of applications that can push forward advances in clinical medicine right now. We also assess the current landscape of Clinical NLP tools and techniques on our adjoining public GitHub site, which can be updated by the research community as clinical NLP technologies evolve [17].

## Practical Consideration #1: Are Data and Compute Infrastructure Ready for NLP?

“Garbage in, garbage out” refers to low-quality data, or “garbage,” that can result in misinformation. It was first used by US Army scientists to provide the intuition that computers cannot think for themselves, and that “sloppily programmed” inputs inevitably lead to incorrect outputs. Although this saying is over a half century old, it applies even more today, when powerful computers can record large amounts of data that are not fit for the intended use in a short amount of time.

Key questions that will help to determine NLP readiness of a new clinical corpus includes the following: (1) Are notes and note metadata reported in a timely way and with reasonable quality? (2) Is the unstructured free-text data ready for NLP techniques (eg, can the data be used to extract clinical concepts with an accuracy that is fit for the indented use)? And (3) Are the NLP algorithms feasible to execute in the production environment?

Assessing the quality of textual data—or “Data Readiness”—confronts on the problem of data quality by providing empirical findings about syntactic and semantic aspects of a clinical corpus as well as the associated note metadata such as patient identifiers, the date and time of the note, and the type of note. We define “quality” within the context defined by Kahn et al [18] where three dimensions are considered, including plausibility, conformance, and completeness. The results of a Data Readiness assessment help to predict the difficulty of building an NLP system for those data. The quality of free-text data can vary significantly between different note types within the same or across different electronic medical record (EMR) systems. For example, discharge summaries typically contain complete sentences and clearly demarcated sections. By contrast, intensive care unit (ICU) progress notes typically contain large quantities of digits that are not explicitly labelled as to whether they are vital signs, ventilator settings, or any of the many other quantitative measures that are monitored in critically ill patients. ICU progress notes also frequently contain large amounts of information in just one or two grammatically unstructured sentences. Ambulatory progress reports can range from a few sentences to longer documents with standardized formats.

In some cases, data sets that do not initially appear to be ready for NLP on an intended task can be further processed or sampled so that the data are more amenable to their intended use. For example, a data source can be preprocessed to remove notes that do not fit predetermined plausibility criteria, such as the known range of system availability to identify notes that have a plausible date, or notes that do not have an indicated date. However, this may not always result in data that are ready for NLP; in these cases, investigators should work with an organization leadership to improve data collection before undertaking an NLP project.

The institutional nuances of EMR clinical documentation processes require clinical NLP systems developed at other institutions to be customized to a new local data set. This uses specific preprocessing steps related to the provenance and structure of the source data. In prior work funded by the Agency for Healthcare Research and Quality that was based on the Rheumatology Informatics System for Effectiveness (RISE) registry, we found that simple summary statistics on note length in characters and words (“tokens”) were helpful to assess the quality of clinical notes from rheumatology practices across the United States [12]. The RISE registry began operation in 2014, and the free-text extraction covered the period between 2014 and 2018. It combines data from over 260 ambulatory outpatient rheumatology practices that collectively use more than 20 different EMR products. To assess the data readiness of RISE for health services research and to better understand the epidemiology of chronic rheumatic diseases, we first used note metadata. For example, we calculated the number of unique clinical notes recorded by year, as indicated by the time stamp of the patient note. Unique notes were determined by each entry of a textual document within the RISE database. We found that many notes had an invalid timestamp, with dates as early as 1800 and as far out as 8018. This suggested an opportunity to improve the quality of these data. We also found that simple summary statistics describing the textual data helped determine the potential informativeness of RISE for scientific and practical applications. Table 1 suggests that RISE contains many relatively short patient notes (mean of 34.57 tokens in 2018) as well as some longer, more traditional patient notes and letters (SD 203.01 tokens). These types of summary statistics are an important first step in NLP data readiness assessments.

**Table 1:** Mean, SD, minimum, mode, and maximum note length^a^ and word count^b^ for free-text patient Rheumatology Notes submitted to the American College of Rheumatology's data registry, by year.

Year	Note count	Length of note					Word count			
		Mean (SD)	Min^c^	Mode	Max^d^	Mean (SD)		Min	Mode	Max
2010	891,837	96 (353)	1	17	18,774	16 (54)		—^e^	2	2549
2011	1,238,711	128 (554)	4	17	40,295	20 (80)		1	2	5713
2012	2,412,737	118 (559)	3	19	23,370	18 (81)		1	2	3496
2013	3,409,806	120 (597)	3	19	23,921	18 (87)		1	2	3567
2014	5,394,083	209 (1069)	1	19	614,356	31 (160)		—	2	107,498
2015	7,715,894	211 (1547)	1	19	2,179,227	31 (224)		—	2	375,620
2016	9,812,735	233 (1356)	1	19	425,503	34 (186)		—	2	75,844
2017	11,685,000	242 (1468)	1	19	570,721	35 (204)		—	2	100,311
2018	5,301,039	239 (1415)	1	19	192,570	35 (203)		—	2	31,852
Total	50,222,840	205 (1271)	1	19	2,179,227	30 (180)		—	2	375,620

^a^Please note that 2018 is a partial year. Note length is indicated by non–whitespace characters and symbols.

^b^Word count was estimated after deidentification of the Rheumatology Informatics System for Effectiveness corpus.

^c^Min: minimum.

^d^Max: maximum.

^e^Not available.

To assess the readiness of data for specific linguistic analysis tasks, such as part-of-speech tagging or named-entity recognition, there are a variety of other descriptive statistics based on corpus linguistics that can be used to assess the quality of textual data. Some of these focus on gross characteristics of the data, such as the extent to which documents have clearly identifiable sections and the nature of the data in those sections. For example, lists such as diagnoses and medications usually have relatively clear boundaries, while family and individual medical histories may not. The presence or absence of sentence boundaries, as well as the length of sentences, are also important predictors of the effort required to build high-performing language processing tools. Other descriptive statistics assess textual data on the level of individual words. For example, textual genres with high levels of repeated word use (eg, fever and pain) can be easier to process than textual genres with high levels of words that only appear once (eg, misspellings and typographic errors).

In addition to data that are ready for NLP, automated information extraction algorithms require infrastructure that will allow for the efficient processing of large volumes of new patient notes. There must be discussions at the design phase of the project to ensure that any research products can be operationally tested, and if warranted, translated to operational infrastructure. It is also important for the product to be updated and maintained if being used longitudinally with routine updates of notes.

If a project has no feasible pathway to operationalize the NLP system for real-world decision support, it might be possible that new resources, including institutional computing infrastructure, could be recommended and acquired.

## Practical Consideration #2: What are the Incentives for Adopting the NLP System?

Key questions that will help determine if the proper incentives are in place to support a Clinical NLP system are as follows: (1) will the NLP help to address an existing clinical need? (2) is there support from clinical leadership for the ongoing use of the NLP system? and (3) is there a financial incentive to adopting the NLP system?

Reporting from structured data has been the mainstay in health care practice for decades. The Sentinel active surveillance system for medical products and Observational Medical Outcomes Partnership (OMOP) initiatives helped to pioneer the use of common data models to support regulatory initiatives [19,20]. Building on OMOP’s common data models, the Observation Health Data Science Initiative’s extension has extended the OMOP schema to incorporate unstructured data with the “NOTE” and “NOTE_NLP” tables. It is likely that EMR databases will become even more powerful for regulatory initiatives when they can jointly leverage various data modalities such as patient notes or images for the purpose of improved patient care. However, in the absence of a specific clinical need that a system is designed to address, and without the proper incentives to use the system, it is unlikely that a system will be adopted for clinical uses such as decision support, regardless of performance on a research task. A successful system for population and precision health must be innovative, pragmatic enough to be deployed in a production environment and directly aligned with organizational incentives and clinical leadership’s priorities. It should support interoperability but also allow for customization to the nuances of different health systems. We discuss some of these challenges in the next section.

In cases where there is little or no organizational incentive to adopt a clinical NLP system, it is unlikely to succeed past the research phase. Therefore, working with leadership to identify the potential value to a health system and finding possible incentives to adopt such a system are important first steps.

## Practical Consideration #3: Feasibility of Implementation and Evaluation

Key questions that will help to determine the feasibility of implementing and evaluating a clinical NLP system include the following: (1) What is the task (ie, clinical need) that this system seeks to address? (2) Are the clinical concepts of interest captured in structured data? If so, are there limitations to what can be extracted? (3) If NLP is justified, are simple NLP techniques enough or are more complex algorithms warranted? (4) Can the Clinical NLP tool be developed and implemented in a reasonable timeline to fulfill stakeholder needs? (5) What are potential sources of bias, considering factors such as the NLP approach, the data used to train the NLP tool, and the population to which it is applied?

An important early consideration is regarding the target population. In cross-validation over random folds, models are trained and tested over the same population. However, in practice, models are often developed in a training data set but applied to novel data that may originate from a different underlying population of patients or clinicians. Differences in clinical practice and workflow patterns, as well as lack of homogeneity in clinical language (as described above), can have large impacts on the transportability of models from where they were developed to a given target population. This is important to factor into the training assessment (eg, being aware of overfitting) and possibly also into model development. If an available external test set exists that represents the target population, it should be tested as part of the model development process to ensure that the NLP tool is portable and externally valid. Ideally, performance metric reporting should be required for all tools meant to be transportable outside of their training corpus.

There are multiple strategies for mitigating bias and improving portability of NLP tools. One source of bias may arise from the specific type of note used to develop a model; for example, an NLP tool developed only on ICU notes, pathology reports, or notes within a certain specialty may not generalize to other note types or clinical settings. Therefore, different note types should be incorporated into the training corpus, if in fact, the target corpus is intended to involve multiple types. Additionally, as previously described, incorporating a secondary data set that represents the target population for testing, apart from the primary data set used for training, can help ensure that the model is transportable and performs well across health care settings, EMRs, and patient populations.

To evaluate model performance, one must decide at which level the assessment should occur, that is, at the mention, document, or patient level. NLP models can be evaluated by their precision (positive predictive value), recall (sensitivity), specificity, *F*_1_-score (harmonic mean of precision and recall) and overall accuracy compared to a “gold-standard” test set of reviewed text [5,9]. However, the text-specific evaluation may not be as important as the document or even patient-level performance, especially if multiple mentions per patient occur, or structured data fields are being incorporated into the evaluation in conjunction with NLP annotations. Therefore, although at the mention level, the NLP model may correctly identify a patient as positive, it may be that it is only when combined with the additional information (other mentions, lab results, etc) that the output and model performance are clinically important.

As important as model performance at the time of development is, more crucial may be the model performance over time. Validation of NLP models is key both retrospectively and prospectively, as data change longitudinally. It is important for models to be evaluated continually to determine whether they should be fine-tuned and updated, and whether any biases exist. For example, this may involve updating rule-based code to reflect changes in language representation or reevaluating or redeveloping deep learning–based NLP models.

If a clinical NLP system does not address a known and ideally high-priority clinical need, it is less likely to be adopted into practice. However, it may be possible to adapt the system to address a need identified by organizational leadership. If it does not initially show good performance, continued development may help to improve the clinical systems accuracy, especially if linguistic annotation data can be generated and made available for training a better model. Lastly, in some cases where additional expertise can be used for a project, it may be possible to meet a project deadline that would otherwise not be possible. Importantly, having a strategy, including a business plan, for maintaining deployed models is important to ensuring that their clinical application is sustained.

## The Potential Role of NLP in Real-World Decision Support

NLP has the potential to improve population health outcomes in the United States. For example, in the inpatient care setting, NLP systems could reliably identify individuals with symptoms of diarrhea reported in progress notes and feed these data into algorithms for *Clostridioides difficile* testing. Inpatients with falls documented in clinical notes could trigger alerts to discontinue sedatives or narcotics. In the outpatient setting, NLP can be used to assess the severity of a disease or a postoperative complication. The NLP of free-text patient notes also creates opportunities for national, routine quality and performance measurement, which can support improvement in the value of health care delivered to patients at highest risk for poor outcomes [9-12,21-23]. As health systems across the United States move toward whole-person care paradigms, NLP systems can also be used to identify important clinical decision support factors that are undercoded or altogether absent from structured data sources in patient records, such as the presence of behavioral, psychosocial, and economic risk factors.

Predictive analytics is another area where incorporating clinical text has the potential to improve population health [5-7,24]. Most models for population-level risk stratification that use health care data have exclusively relied on structured data, but several groups have demonstrated that in certain domains, adding information from clinical text can improve performance. Studies in this area reflect a wide range of tasks from predicting hospital readmissions to identifying patients at risk for suicide [2-13,17,21,22,24,25]. Such models can be used operationally to more accurately target a subset of a population for specific interventions designed to address modifiable risk factors.

Applications of NLP to streamline and facilitate quality and safety reporting are also emerging [9-12]. Federal reporting of quality and safety measures often places considerable burden on clinicians, sometimes requiring duplicate entry of similar concepts in the text of clinical notes as well as in structured fields that can be queried to calculate performance. Reliable extraction of relevant information from clinical notes would not only alleviate burdensome data entry but also greatly expand the types of concepts included in reporting programs. For example, guidelines in rheumatology support the routine collection of disease activity scores for patients with rheumatoid arthritis, but not all EMRs have structured fields to input these scores. Electronic quality measures that extract this information automatically from structured fields might miss scores that are documented only in clinical notes. NLP could be used to extract these scores and improve the validity and reliability of such quality measures[9-12].

While these and other applications of NLP have the potential to improve health care and population health, the successful deployment and dissemination of these applications has been limited. Given these barriers, how should the field move forward? In addition to our three considerations, we think it is critical that multiple stakeholders provide input from the start of NLP projects. Practicing clinicians can ensure the focus of the work is clinically relevant, fulfills an unmet need, and is aligned with current clinical workflows; clinical informaticians can provide insight into whether data systems are available to scale valid NLP algorithms, and health care administrators can lend insight into IT resources required and the feasibility of scaling and sustaining systems. Until there is stakeholder alignment and investment in a project, impact and scalability are likely to be limited. Similar to many new technologies in medicine, alignment often requires the development of the NLP program as a value proposition that either clearly impacts operational efficiency, revenue, quality and safety, or patient outcomes. Moreover, stakeholders need to be integrated into the software development life cycle to ensure the product’s ongoing implementation is successful.

## Conclusion

The analysis of unstructured free-text patient data enables new ways in which scientific questions can be studied and health care can be delivered. Although such uses are promising, leveraging the clinical text data collected in the EMR and using these data in health care operations are not without substantial caveats. Opportunities to better align state-of-the-art systems developed by researchers to support the measurement of patient-reported outcomes and to support high-quality health care delivery can likely lead to improved outcomes. With a focus on designing practical applications that are aligned with clinical requirements and organizational incentives, the considerations listed here can be used to design a project-specific checklist for a variety of stakeholders. We also summarized the procedures for considering appropriate use of NLP in health and survey the current landscape of Clinical NLP tools. To support future work in this area, we have provided software and data set summaries, license, and other access requirements on our adjoining GitHub site, which we hope will serve as a continuously updated resource for the research community as technologies evolve.

## Abbreviations

EMR: electronic medical record
ICU: intensive care unit
NLP: natural language processing
OMOP: Observational Medical Outcomes Partnership
RISE: Rheumatology Informatics System for Effectiveness
